# A novel olfactory threshold test for screening cognitive decline among elderly people

**DOI:** 10.1371/journal.pone.0254357

**Published:** 2021-07-12

**Authors:** Sun Mi Kim, Hye Ri Kim, Hyun Jin Min, Kyung Soo Kim, Jae-Chan Jin, Doug Hyun Han

**Affiliations:** 1 Department of Psychiatry, Chung-Ang University College of Medicine, Seoul, Republic of Korea; 2 Department of Otorhinolaryngology, Head and Neck Surgery, Chung-Ang University College of Medicine, Seoul, Republic of Korea; 3 Department of Social Welfare, Graduate School of Soongsil University, Seoul, Republic of Korea; Nathan S Kline Institute, UNITED STATES

## Abstract

Olfactory impairment is associated with dementia and is a potential early biomarker of cognitive decline. We developed a novel olfactory threshold test called *Sniff Bubble* using rose odor-containing beads made with 2-phenylethyl alcohol. We aimed to define cut-off scores for this tool to help identify cognitive decline among elderly people. In total, 162 elderly people (mean age ± SD: 73.04 ± 8.73 years) were administered olfactory threshold and neurocognitive tests. For analyses, we divided the participants into two groups based on cognitive functioning, namely cognitive decline (n = 44) and normal cognition (n = 118) groups. The *Sniff Bubble* and YSK olfactory function test for olfactory threshold and the Structured Clinical Interview for DSM-5 Disorders-Clinician Version and Korean version of the Consortium to Establish a Registry for Alzheimer’s Disease assessment packet for neurocognitive functioning were used. We used K-means cluster analyses and receiver operating characteristic (ROC) analyses to identify the most appropriate cut-off value. We established a positive correlation between the *Sniff Bubble* and neurocognitive function test scores (*r* = 0.431, p < 0.001). We defined the cut-off score, using the ROC curve analyses for *Sniff Bubble* scores, at 3 and higher with an area under the curve of 0.759 (p < 0.001). The *Sniff Bubble* test can adequately detect cognitive decline in elderly people and may be used clinically as the first step in the screening process.

## Introduction

Dementia is a major health and economic burden that affected approximately 47 million patients worldwide in 2015, and this number is estimated to nearly double every 20 years (65.7 million in 2030 and 154 million in 2050) [1, 2]. Although dementia is currently incurable, early screening offers several advantages. Specifically, it informs and facilitates quicker decision-making and planning by patients with dementia and their caregivers. Furthermore, pharmacological treatment, including cholinesterase inhibitors or the N-methyl-D-aspartate receptor antagonist (memantine), contributes toward slowing dementia progression [3]. Moreover, it is important to protect patients with dementia from associated risks involving self-neglect and money management, as well as neuropsychiatric symptoms such as agitation, depression, and psychosis [4]. Given the high global burden of dementia, as well as the lack of therapeutic and preventive interventions, there is a need for studies on early dementia screening.

The association between olfactory impairment and dementia is well established. Olfactory dysfunction is among the earliest symptoms of Alzheimer’s disease (AD); therefore, it is a potential biomarker for early preclinical screening of cognitive decline [5, 6]. Dysfunctions in major olfactory functional domains, including odor detection, identification, and discrimination, appear prior to cognitive decline in patients with AD [7]. Patients with AD have been shown to present significantly greater olfactory impairment than individuals with normal aging [8]. Moreover, olfactory dysfunction has been reported in individuals with mild cognitive impairment (MCI) [9, 10]. Furthermore, previous longitudinal studies have reported the potential utility of olfactory function testing of patients with MCI for the screening of likely progression to dementia [11, 12]. According to a review of studies on the association between sensory deficits and AD, olfactory dysfunction is more associated with preclinical AD compared to other visual and auditory deficits [13]. Odor identification impairment predicts the transition to MCI in a cognitively normal group and to AD in patients with MCI, and this also shows a significant association with other biomarkers of AD [13]. Another review on the olfactory pathology in preclinical AD patients suggested that olfactory impairment in early AD may support the infectious hypothesis of AD [14]. The infectious hypothesis proposes that infection (such as viral, bacterial, or fungal infection) is associated with amyloidogenesis and that neuroinflammatory reactions occur first in the peripheral olfactory system outside the blood-brain barrier and then spread into the central olfactory system [14]. This hypothesis suggests the potential of an olfactory test as a non-invasive screening tool for early AD [14]. In addition, in late-life depression, another group at high risk of AD, patients with impaired odor identification had a higher risk of AD progression than those with intact odor identification [15]. Therefore, olfactory function tests can be used to identify cognitively normal adults at risk of MCI or dementia before the onset of clinically detectable cognitive decline.

The neuropathology of AD is characterized by neural loss, intracellular neurofibrillary tangle deposition, and amyloid-β plaques in the brain [16]. Various anatomic structures in the peripheral and central olfactory system in patients with AD can be affected, including the olfactory epithelium, olfactory bulb, entorhinal cortex, and hippocampus [8]. Therefore, patients with AD can present with various olfactory functional deficits, including those in the detection threshold (detectable odor molecular concentration), discrimination function (the ability to distinguish one odor from others), and identification ability (the ability to associate odor molecules with related words or images) [8, 9, 17].

These previous findings indicate that olfactory function tests could allow AD screening and the detection of early cognitive decline. In a clinical setting, simple and effective tools that require <5 min to administer are considered the most suitable for dementia screening [18]. Therefore, we aimed to develop a simple olfactory threshold test to screen for cognitive decline that could help identify elderly individuals at risk for MCI or dementia. We adopted an olfactory threshold test given that it is simpler, is less time consuming, and requires less equipment compared to odor discrimination and identification tests. Odor identification is the most studied biomarker of MCI and dementia compared to odor threshold and discrimination [12]. However, the University of Pennsylvania Smell Identification Test (UPSIT), the most commonly used odor identification test, which uses 40 scents, takes too long to administer and is complicated to use as a screening test for the elderly [19]. Furthermore, odor identification can be affected by differences in region, culture, and individual experiences [20]. Thus, it is difficult to generalize the results across cultures and ages. We developed an odor threshold test for use as a dementia screening tool that takes less than 5 min to administer, is easy to use in the elderly, and can be used cross-culturally. Compared with cognitively healthy individuals, patients with AD have been shown to have higher olfactory thresholds (lower sensitivity) [21, 22]. In addition, the impairment level is correlated with AD severity [22]. Based on these previous findings, we hypothesized that individuals with cognitive decline, as measured by neurocognitive tests, would present olfactory deficits. We aimed to develop a clinically applicable screening tool that could measure the olfactory threshold. Moreover, we aimed to determine its cut-off score that could identify individuals with cognitive decline who require further neurocognitive assessment.

## Materials and methods

### Participants

This cross-sectional study was conducted between August 2018 and May 2019 at the Department of Psychiatry of the Chung-Ang University Hospital in Seoul, Korea. We enrolled patients with a pre-existing MCI or dementia diagnosis, individuals on their first visit for cognitive assessment, and individuals who responded to a hospital bulletin advertisement.

The inclusion criteria were (a) age > 50 years and (b) being aware of the study and having provided informed consent. The exclusion criteria were as follows: (a) any past or current neurologic (e.g., brain tumor, epilepsy, and Parkinson’s disease) or psychiatric diseases (e.g., major depressive disorder, bipolar disorder, or schizophrenia) other than MCI and dementia (major neurocognitive disorder) based on the Structured Clinical Interview for DSM-5 Disorders-Clinician Version (SCID-5-CV) [23]; (b) any past or current diagnosis of dementia other than Alzheimer’s disease dementia (e.g., vascular dementia, Parkinson’s disease dementia, and Lewy body dementia); (c) head trauma or stroke history; (d) acute rhinitis or sinusitis, active asthma, or a history of obstructive nasal disease or nasal sinus surgery; (e) communication difficulties resulting from severe hearing impairment or aphasia; and (f) failing to understand the study protocol and objectives. This study was approved by the Institutional Review Board of the Chung-Ang University Hospital (Approval number: 1811-001-309), and each participant and their caregiver (spouse or adult child) provided written informed consent.

Among 186 initially screened elderly people, we excluded two individuals who did not understand the study protocol and objectives, three who had communication difficulties, three who had a history of nasal polyp or nasal sinus surgery, six who had other neurologic or psychiatric diseases (Parkinson’s disease and major depressive disorder), and five who had a history of head trauma or stroke. The remaining 167 individuals were enrolled in this study. Among them, five participants failed to complete the neurocognitive test; thus, 162 participants were included in the final analysis.

### Screening tool development

**Fig 1:**
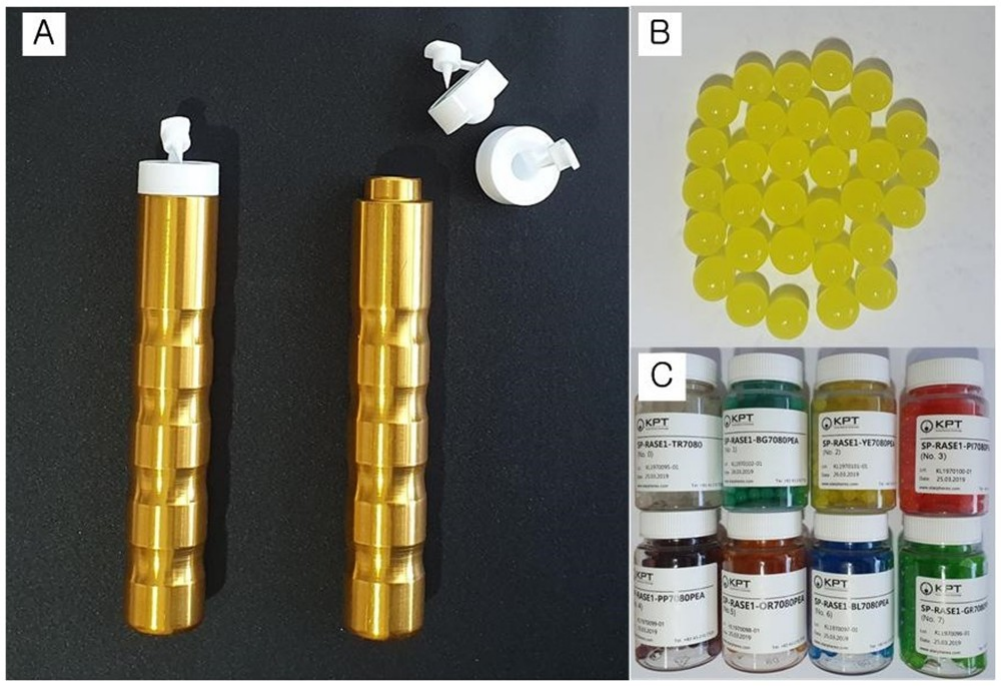
A novel olfactory threshold test kit, *Sniff Bubble*. (A) Aluminum-made bodies and disposable plastic capsule containers. (B) Rose odor-containing beads made with 2-phenylethyl alcohol (PEA). (C) Eight types of beads with the seven PEA concentrations ranging from 10%–0.16% and distilled water.

We developed a novel olfactory threshold test kit called *Sniff Bubble* (Fig 1). This test kit was made using 2-phenylethyl alcohol (PEA), which has a rosy smell that is considered ideal for estimating olfactory acuity [24, 25]. We used odorless distilled water as the blank stimulus and as the diluent for the threshold series (consisting of seven concentrations ranging from 10% to 0.16%, with scores ranging from 1 [highest concentration] to 7 [lowest concentration]) (Table 1).

**Table 1:** Eight types of beads with seven PEA concentrations and volume of distilled water.

Number of beads (Score)	PEA concentration (%)	Volume of distilled water (ml)	Volume of PEA (ml)	Total volume (ml)
0 (distilled water)	0	500.00	0	500
1	10.00	450.00	50.00	500
2	5.00	475.00	25.00	500
3	2.50	487.50	12.50	500
4	1.25	493.75	6.25	500
5	0.63	496.87	3.13	500
6	0.31	498.44	1.56	500
7	0.16	499.22	0.78	500

PEA, 2-phenylethyl alcohol

We made beads using each of the seven PEA concentrations and distilled water. A disposable plastic capsule container was mounted on an aluminum-made body and beads were placed on it using tweezers. We then closed the lid of the capsule container, which was equipped with a pointed portion that released the fragrance as the beads burst.

The Sniff Bubble test is a newly developed olfactory detection threshold test that uses odor-containing beads. Compared to the conventional Sniffin’ Sticks test using felt-tip pens, this test is simple to use and takes less time. The YSK olfactory function test used as a comparative test in this study is a modified test that added odors familiar to Koreans to the Korean Version of the Sniffing’ Sticks (KVSS-II) test. The KVSS-II test is bulky, with 102 test pens, takes more than 30 min to complete, and requires professional staff to perform the test [26]. The YSK olfactory function test has reduced the number of test pens to 84, and the test time has been shortened to approximately 20 min, but it is still too long and complicated to administer to elderly individuals with cognitive impairments [27]. The Sniff Bubble test simplifies the tool, making it easy to carry and use. The average (mean ± standard deviation) test time of the Sniff Bubble test (5.00 ± 1.51 min) is less than that of the YSK olfactory function test (YSK total: 20.4 ± 5.3 min, YSK threshold test: 6.41 ± 2.71 min) [28].

The Sniffin’ Sticks test is an accurate scoring tool for the evaluation of the olfactory threshold of olfactory function and adjusts for confounders such as guessing, olfactory adaption, and training using the seven turning points. The Sniff Bubble test has poor confounder control, but since this test was developed as a screening tool, it is more important that the tester can perform it easily and quickly. In fact, it was ultimately developed with the aim of using this test not only in primary medical institutions and public health care centers but also to enable self-diagnosis by patients and families at home.

### Clinical evaluation and procedures

#### *Sniff Bubble* test

An olfactory test technician conducted the screening session and olfactory function tests for each participant in a dedicated, well-ventilated laboratory. For increased accuracy, we conducted the olfactory test in a place lacking other scents, odors, and wind. Initially, the examiner instructed the participant to smell the distilled water beads. In case the participant detected a smell, the examiner recorded the reported description of the smell. This test was necessary for assessing the odorless test environment and verifying that the participants were suitable for the test. Subsequently, the examiner asked the participants to smell the fourth-strongest PEA concentration and state its fragrance. In case the participants answered that they could detect the fragrance, they were asked to smell progressively weaker odor concentrations. In contrast, if the participants answered that they could not recognize the fragrance, they were asked to smell progressively stronger odor concentrations. Regarding odor presentation, scented beads were burst before placing them 2 cm in front of both nostrils for about 3 s. The threshold was defined as the lowest PEA concentration at which the participant gave a correct response. The scores ranged between 1 and 7, with a lower score indicating a higher olfactory threshold or lower olfactory detection ability.

#### YSK olfactory function test kit

To assess the correlation between *Sniff Bubble* scores and those of existing olfactory function tests, we used the YSK olfactory function test kit (RHICO Medical Co., Seoul, Korea) [27]. This kit was recently developed using odorants culturally familiar to Koreans. It comprises of three olfactory function subtests, i.e., olfactory PEA threshold, odor discrimination, and odor identification. The odors are presented in felt-tip pens with tampons filled with fluid odorants. To present the odor, the examiner removes the cap and places the tip of the pen at about 2 cm in front of both nostrils.

We evaluated the olfactory threshold using 12 PEA concentrations in a geometric series. Here, the participant was asked to identify the pen containing the odorant. In this test, the scores range from 0 to 12. In the odor discrimination subtest, the 12 sets of three pens each were randomly presented. Among the three pens, two contained the same odorant while the third contained a different odorant. The participant was asked to identify the pen with a different smell. The score after presenting all twelve sets ranged from 0 to 12. In the odor identification subtest, 12 odors common to Koreans were presented to the participants. The participants were allowed to sample each odor as required and select one of four descriptors for each odor. The total score ranged from 0 to 12. The three subtest results were summed to give the total score.

#### Neurocognitive test

Two psychiatrists screened the participants for MCI and major neurocognitive disorder using the SCID-5-CV [23]. The Korean version of the Consortium to Establish a Registry for Alzheimer’s Disease assessment packet (CERAD-K) was used to assess objective neurocognitive functions. The CERAD investigators in the USA developed a standardized clinical and neuropsychological assessment battery for evaluating patients with AD [29]. AD diagnosis using the CERAD assessment battery is highly accurate [30]. Moreover, previous studies on elderly Koreans have demonstrated the CERAD-K as substantially reliable and valid and comparable to the American version [31, 32]. The CERAD-K consists of the following items: Verbal Fluency, Modified Boston Naming, Mini-Mental State Examination, Word List Memory, Constructional Praxis, Word List Recall, Word List Recognition, Constructional Praxis Recall, Trail Making Test, and Stroop Test. We obtained each participant’s subscores, total score (from 0 to 100), and three cognitive function levels after adjusting for age and sex (normal range/significant decline but not dementia level/impaired performance comparable to dementia). Based on the SCID-5-CV and CERAD-K results, the study participants were divided into two groups: (a) the cognitive decline (Cog-D) group, which consisted of participants with MCI or major neurocognitive disorder, and (b) the normal cognition (Normal) group, which consisted of participants with normal cognitive function.

### Statistical analysis

Between-group comparisons of the demographic characteristics were performed using the independent t-test and chi-square test. We determined the correlations of the *Sniff Bubble* scores with YSK olfactory function test scores (detection threshold, discrimination, identification, and total scores) and CERAD-K scores. We performed a K-means cluster analysis to allocate the participants to either the Cog-D or Normal group. Receiver operating characteristic (ROC) curves were used to calculate the sensitivity, specificity, and area under the ROC curve (AUC), as well as to determine the cut-off value for identifying individuals with cognitive decline. The value with the highest sensitivity + specificity was considered the best cut-off point. All statistical analyses were performed using SPSS ver. 19.0 (IBM Corp., Armonk, NY, USA).

## Results

### Sample characteristics

We allocated 162 participants to the Normal (n = 118) and Cog-D (n = 44) groups. There were significant between-group differences in the age (Cog-D: 80.18 ± 7.51 years, Normal: 70.38 ± 7.61 years; t = -7.320, p < 0.001; Table 2). Regarding the education level, the Cog-D group had significantly more participants who reported illiteracy and primary school graduation, whereas the Normal group had more participants who reported college, university, and graduate school graduation (χ^2^ = 6.583, p < 0.05). There were no significant between-group sex differences.

**Table 2:** Demographic characteristics of the study population (N = 162).

	Range	Normal (n = 118)	Cog-D (n = 44)	Statistics (t/χ^2^, p-value)
Age (years)	57–96	70.38 ± 7.61	80.18 ± 7.51	-7.320*
Sex (male/female)		28/90 (23.7%/76.3%)	9/35 (20.5%/79.5%)	0.195, 0.659
Level of education				6.583**
Illiteracy and Primary school		41 (34.7%)	23 (52.3%)	
Middle and High school		56 (47.5%)	19 (43.2%)	
College/University and Graduate school		21 (17.8%)	2 (4.5%)	
CERAD-K	0–100	69.96 ± 13.45	23.77 ± 13.59	19.386*
YOF				
Threshold	0–12	2.60 ± 1.78	.75 ± 1.18	6.366*
Discrimination	0–12	6.50 ± 2.16	3.82 ± 2.15	7.040*
Identification	0–12	9.70 ± 2.94	4.36 ± 2.91	10.313*
Total	0–36	18.80 ± 5.17	8.93 ± 4.78	11.030*
*Sniff Bubble*	0–7	4.14 ± 1.61	2.36 ± 1.92	5.481*

*p<0.001;

**p<0.005

Cog-D, cognitive decline group; CERAD-K, Korean version of the Consortium to Establish a Registry for Alzheimer’s Disease; Normal, normal cognition group; PEA, phenylethyl alcohol; YOF, YSK olfactory function test

### Analyses of the correlation of *Sniff Bubble* scores with YSK olfactory function test and CERAD-K scores

**Fig 2:**
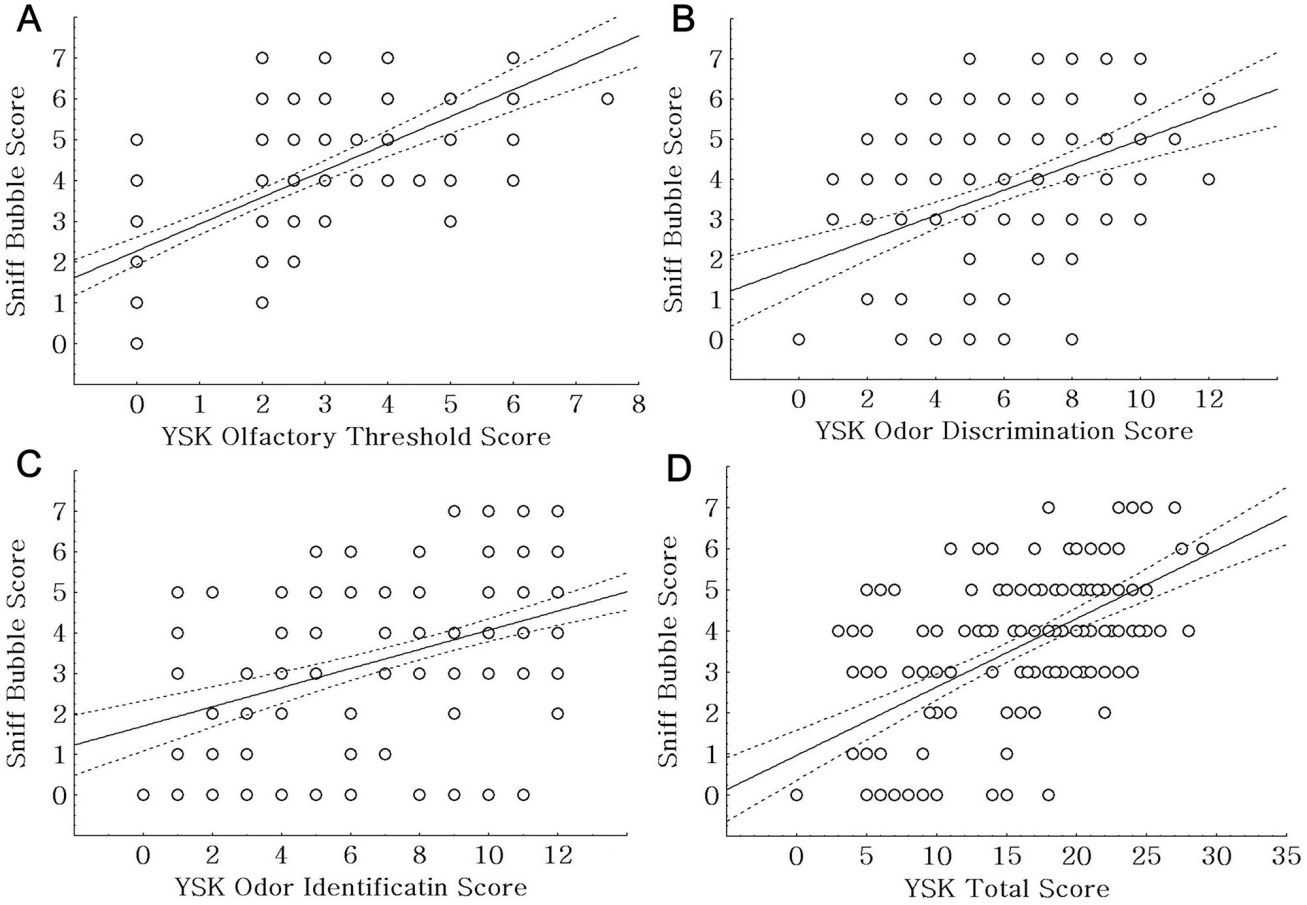
Analyses of the correlation between the *Sniff Bubble* and YSK olfactory function test scores. (A) There was a positive correlation between *Sniff Bubble* and YSK olfactory threshold test scores (*r* = 0.646, p < 0.001). (B) There was a positive correlation between *Sniff Bubble* and YSK odor discrimination test scores (*r* = 0.414, p < 0.001). (C) There was a positive correlation between *Sniff Bubble* and YSK odor identification test scores (*r* = 0.477, p < 0.001). (D) There was a positive correlation between *Sniff Bubble* and YSK olfactory function test total scores (*r* = 0.597, p < 0.001).

**Fig 3:**
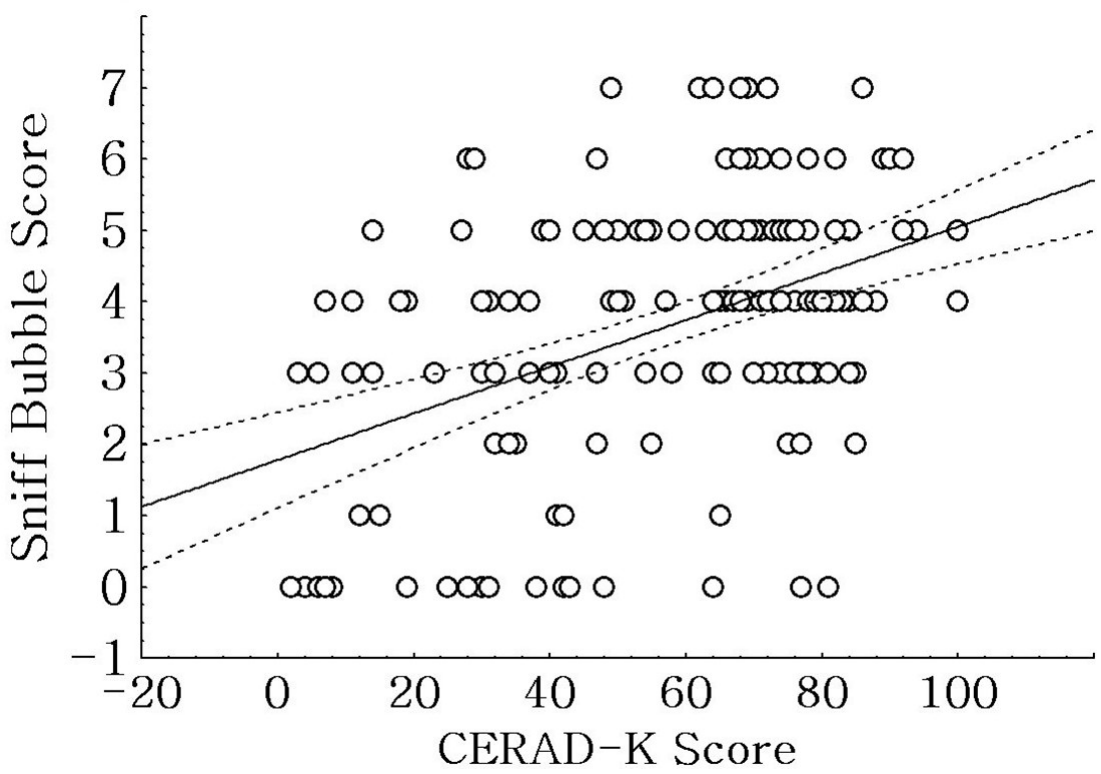
Analysis of the correlation between the *Sniff Bubble* and the neurocognitive test scores. There was a positive correlation between the *Sniff Bubble* and the Korean version of the Consortium to Establish a Registry for Alzheimer’s Disease assessment packet (CERAD-K) scores (*r* = 0.431, p < 0.001).

The *Sniff Bubble* score was positively correlated with the YSK olfactory threshold test score (*r* = 0.646, p < 0.001), YSK odor discrimination test score (*r* = 0.414, p < 0.001), YSK odor identification test score (*r* = 0.477, p < 0.001), and YSK olfactory function test total score (*r* = 0.597, p < 0.001; Fig 2). Moreover, the *Sniff Bubble* score was positively correlated with the CERAD-K score (*r* = 0.431, p < 0.001; Fig 3).

### Cluster analysis of the study sample for the *Sniff Bubble* score

In the K-means cluster analysis of the 162 participants, the highest (7) and lowest (0) *Sniff Bubble* scores were selected as initial seeds (centroids of respective groups). The final centroid and standard deviation (mean ± SD) of the *Sniff Bubble* scores of the Cog-D and Normal groups were 1.74 ± 1.34 and 4.85 ± 0.91, respectively. The between-group final Euclidean distance was 3.108. After K-means cluster analysis, we allocated the 162 participants into the Normal (100) and Cog-D groups (62).

### Receiver operating characteristic curve analysis of the *Sniff Bubble* score

**Fig 4:**
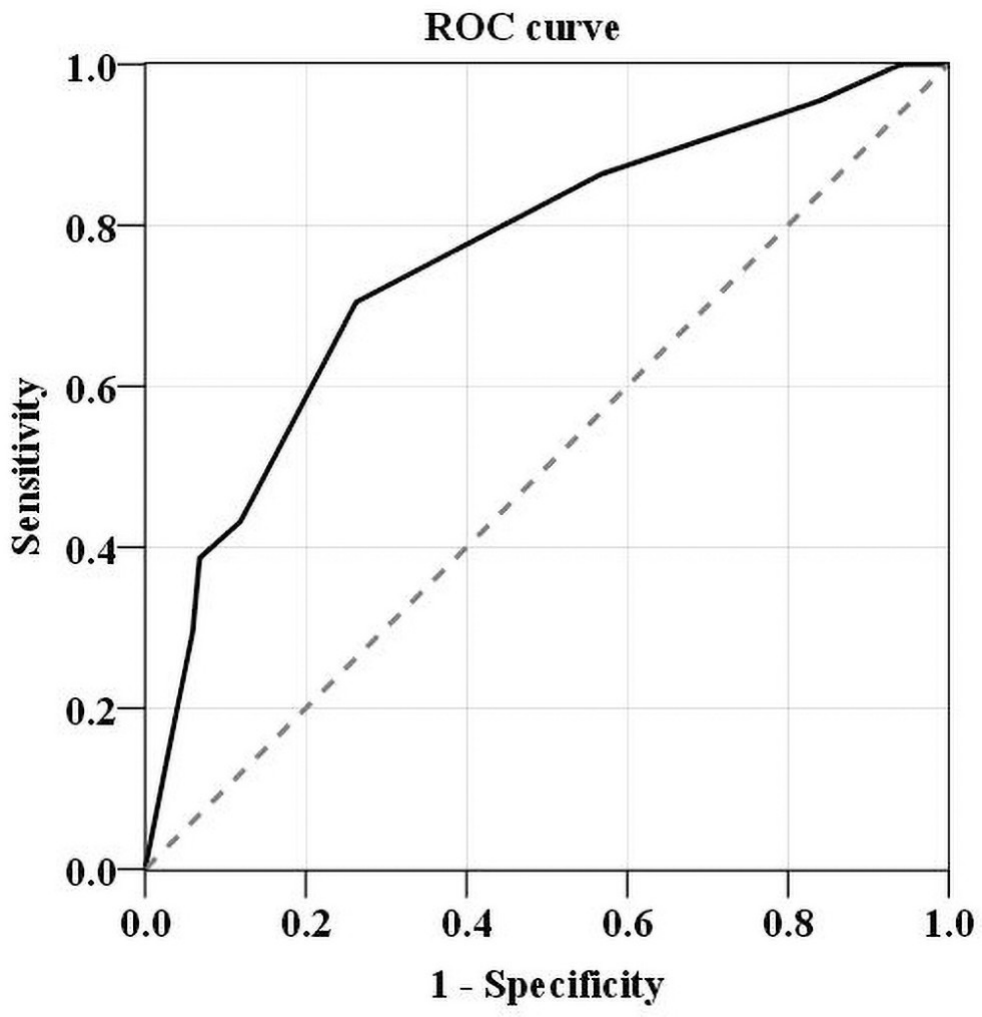
Receiver operating characteristic (ROC) curves for the *Sniff Bubble* scores. Area under the curve (AUC) for the *Sniff Bubble* score was 0.759 (95% confidence interval: 0.673–0.844, p < 0.001).

Among the 162 participants, the cut-off *Sniff Bubble* score in the Cog-D group was 3 with an AUC of 0.759 (95% CI: 0.673–0.844, p < 0.001) (Fig 4). As shown in Table 3, the sensitivity + specificity value was the highest for *Sniff Bubble* scores of 3.

**Table 3:** Sensitivity and specificity of the *Sniff Bubble* scores.

Cut-off value	True positive	False positive	False negative	True negative	Positive predictive value	Sensitivity	Accuracy	Negative predictive value	Specificity	Precision	Sensitivity + specificity
2	104	25	14	19	0.806	0.881	0.759	0.576	0.432	0.806	1.313
**3**	**87**	**13**	**31**	**31**	**0.870**	**0.737**	**0.728**	**0.500**	**0.705**	**0.870**	**1.442**
4	51	6	67	38	0.895	0.432	0.549	0.362	0.864	0.895	1.296

## Discussion

In this study, we developed a novel olfactory threshold test kit called *the Sniff Bubble*. This kit reliably differentiates between cognitive decline and normal cognition in elderly people. This kit, which used rose odor-containing beads made with PEA, was easy to use and simple enough for assistants to use with minimal training. The cut-off *Sniff Bubble* score for cognitive decline was set at 3. A high olfactory threshold measured at a ≤ 3 *Sniff Bubble* score may be useful as a cognitive decline indicator. Therefore, this tool could be sufficient for detecting cognitive decline in elderly people and could be used as an initial screening tool for individuals with cognitive decline who require further cognitive function tests. In a recent study evaluating the usefulness of the YSK olfactory function test for diagnosing dementia, the YSK odor threshold test showed good sensitivity (0.725) and specificity (0.837) in distinguishing between MCI and dementia [33]. The YSK odor identification test showed good sensitivity (0.785) and specificity (0.654) in distinguishing the normal cognition group from the MCI and dementia groups [33]. The UPSIT showed higher sensitivity (0.852) and specificity (0.818) in distinguishing between the normal cognitive group and the dementia group [9]. In the current study, the Sniff Bubble showed good sensitivity (0.737) and specificity (0.728), even though it is a simpler test than the conventional tests.

We observed significant between-group differences in age and education levels. Age is the greatest risk factor for dementia, with its incidence increasing exponentially after the age of 65 [4]. Moreover, approximately 80% of patients with dementia are aged > 75 years [4]. There are complex interactions among age, neuropathology, and comorbid medical conditions. After adjustment for other risk factors and comorbid medical conditions, age *per se* could be a less significant risk factor; however, it remains an important non-modifiable risk factor. Further, there is an association of lower education levels with a higher risk of dementia in late-life [34]. Education level is considered among the potentially modifiable risk factors, including cardiovascular disease, stroke, metabolic syndromes, psychiatric conditions, diet, and lifestyle. Education could contribute to cognitive resilience later in life by building brain reserve through intellectual stimulation [35].

We observed a positive correlation between CERAD-K and *Sniff Bubble* scores. A significantly increased olfactory threshold in patients with AD compared with that in control participants was reported; moreover, it was significantly correlated with the dementia scale score [22]. Additionally, it has been reported that olfactory threshold deficits occur in individuals with early-stage AD and MCI prior to complete clinical manifestation, with further decrease with disease progression [9].

There is an association of olfactory threshold deficits in individuals with cognitive decline with various anatomic structures in the peripheral and central olfactory system. Doty and Richard [17] suggested that changes in detection thresholds arise mainly from peripheral nervous system impairment, whereas changes in odor discrimination and identification are mainly from central nervous system impairment. Olfactory epithelium changes in patients with AD have been reported to cause detection threshold impairments [21, 24, 36]. Moreover, a correlation between the olfactory threshold and central nervous system structures has been reported. Djordjevic et al. [9] suggested that primary olfactory cortex areas, including the piriform and entorhinal cortex, as well as the olfactory nuclei in the amygdala, are involved in odor detection. Using resting-state positron emission tomography, a correlation was reported between olfactory threshold test scores and clusters in the right thalamus and cerebellum [37]. The inconsistency in the findings could be attributed to the use of varying procedures or materials to measure the olfactory thresholds across studies, as well as the use of varying sample sizes [38]. AD neuropathology is characterized by neural loss, neurofibrillary tangles, and amyloid-β plaques affecting related anatomic structures [16]. However, the basic olfactory dysfunction mechanism and its relationship with AD pathology remain unclear. AD rodent models have demonstrated correlations between olfactory dysfunction and both neurofibrillary and amyloid-β deposition patterns within the olfactory system [39, 40]. Moreover, an AD rodent model study reported that amyloid-β deposition occurred in the olfactory bulb before other brain areas [32]. These findings suggest that neurofibrillary and amyloid-β-related mechanisms could contribute to early olfactory dysfunction in AD.

This study has several limitations. In the participants of this study, cognitive decline could have various etiologies, even though our exclusion criteria addressed cognitive decline caused by neurologic diseases, head trauma, and stroke. According to the literature, various anatomic structures from the peripheral and central olfactory systems are affected in patients with AD, which results in olfactory impairment [8]. Therefore, the Sniff Bubble could be more reliable for patients with AD. However, the results of the Sniff Bubble may be confounded with other diseases that are more associated with peripheral dysfunction in the olfactory pathway. In addition, although we excluded participants with cognitive decline caused by neurological diseases such as Parkinson’s disease during participant enrollment, individuals with Parkinson’s disease also exhibit olfactory dysfunction. A meta-analysis comparing olfactory dysfunction between AD and Parkinson’s disease found greater impairment in the olfactory threshold of those with Parkinson’s disease and in olfactory identification in those with AD [41]. Future studies are needed to allow for more accurate neuroimaging-based differential diagnoses of neurodegenerative diseases. In addition, depression has been shown to affect cognitive and olfactory function in the elderly [42]. We excluded patients with depression through a psychiatric interview process (SCID-5-CV) when screening participants, but we could not completely control for the confounding effect of subclinical depressive symptoms. Second, due to time limitations and complexities in assessing elderly people and patients with dementia, we did not measure the test-retest reliability. There is a need for further studies to measure test-retest and inter-rater reliability. Third, since this was a single-center study, it could have had a potential bias in the diagnosis of major neurocognitive disorder. Fourth, since we assessed a hospital-based population, the tool’s usefulness in a community-based sample is unclear. There is a need for multicenter studies on a community-based population to assess the generalizability of our findings.

## Conclusions

In summary, the *Sniff Bubble* can be a useful tool for screening cognitive decline in the elderly. A high olfactory threshold measured at a *Sniff Bubble* score of 3 or less could be indicative of cognitive decline. Moreover, this tool could allow screening of cognitive decline before full clinical manifestation of dementia symptoms. This suggests that this tool could be used by general practitioners, psychiatrists, and neurologists for initial clinical screening of patients with dementia. Further studies are required to assess the diagnostic accuracy of the combined use of *Sniff Bubble* and known cognitive screening tools to improve its accuracy.
